# Variability in visual cortex size reflects tradeoff between local orientation sensitivity and global orientation modulation

**DOI:** 10.1038/ncomms3201

**Published:** 2013-07-26

**Authors:** Chen Song, Dietrich S. Schwarzkopf, Geraint Rees

**Affiliations:** 1 Institute of Cognitive Neuroscience, University College London, 17 Queen Square, London WC1N 3AR, UK; 2 Wellcome Trust Centre for Neuroimaging, University College London, 12 Queen Square, London WC1N 3BG, UK

## Abstract

The surface area of early visual cortices varies several fold across healthy adult humans and is genetically heritable. But the functional consequences of this anatomical variability are still largely unexplored. Here we show that interindividual variability in human visual cortical surface area reflects a tradeoff between sensitivity to visual details and susceptibility to visual context. Specifically, individuals with larger primary visual cortices can discriminate finer orientation differences, whereas individuals with smaller primary visual cortices experience stronger perceptual modulation by global orientation contexts. This anatomically correlated tradeoff between discrimination sensitivity and contextual modulation of orientation perception, however, does not generalize to contrast perception or luminance perception. Neural field simulations based on a scaling of intracortical circuits reproduce our empirical observations. Together our findings reveal a feature-specific shift in the scope of visual perception from context-oriented to detail-oriented with increased visual cortical surface area.

## Abstract (web summary)

Surface area features of developing visual cortices are implicated in visual perception. Song *et al.* measure visual discrimination sensitivity in humans and find that an increase in visual cortical surface area activity is associated with improved discrimination sensitivity and degraded contextual illusions.

## Graphical abstract

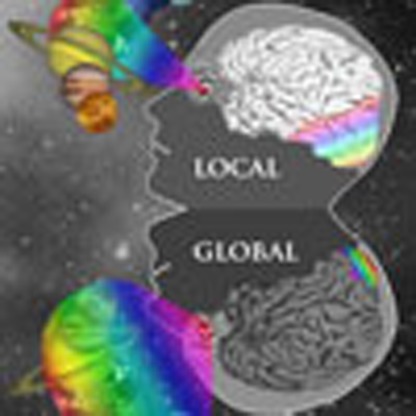

The arealization of cerebral cortex is controlled by an interplay between genetic and developmental factors^1^. In humans, there is a threefold variability across healthy individuals in the retinotopically defined surface area of early visual cortices (V1, V2, V3)^2^. Such variability is substantially greater than the variability in overall cortical size^3^. With recent studies revealing the genetic and perceptual correlates of visual cortical surface area, it has been suggested that the retinotopically defined surface area of early visual cortices may be an important endophenotype determining individual visual experiences^4^,^5^. Nevertheless, exactly how and more importantly, why interindividual variability in visual cortical surface area affects visual perception remains unclear.

Here we investigated two different hypotheses. The retinotopically defined surface area of early visual cortices represents variability across individuals in the amount of cortical surface devoted to the same visual field space^2^. As such, a visual stimulus of the same physical size will activate a larger cortical area and have a larger effective size in individuals with larger visual cortex. One intuitive hypothesis is that visual cortical surface area influences perception through this scaling of effective stimulus size. Two fundamental perceptual properties—sensitivity to visual details and susceptibility to visual contexts—are dependent on the size of a visual stimulus. Increasing the stimulus size generally leads to improved visual discrimination sensitivity^6^,^7^,^8^ but weakened visual contextual modulations (illusions)^9^,^10^,^11^. Thus, individuals with larger early visual cortices may perceive finer visual details, whereas individuals with smaller early visual cortices may experience stronger visual contextual modulations. This first hypothesis suggests a generic change in the scope of visual perception from global, context-oriented to local, detail-oriented as visual cortical surface area increases across individuals.

A second hypothesis is that the visual cortical surface area influences perception not just through the scaling of effective stimulus size, but also through the scaling of intracortical connections. When visual cortical surface area increases, physical constraints may prevent intracortical connections from lengthening at the same rate as the expansion of cortical surface, and different neurons within a neural population activated by a visual stimulation will thus be less interconnected^12^,^13^. This decrease in intracortical connectivity is likely to influence the perception of different visual features differently, as intracortical connections exhibit distinct topologies for different features that the neurons code^14^,^15^. For example, although orientation is coded in an orderly fashion with neurons preferring more similar orientations being more highly connected, visual features such as contrast or luminance does not have an orderly cortical representation where intracortical connectivity covaries with the similarity in feature preference between connected neurons^14^,^15^. It is plausible that an orderly cortical representation of a feature space, where the distance in cortical space reflects the distance in feature space, is required for the scaling of intracortical connections to influence the perception of this visual feature. Our second hypothesis suggests that the perceptual changes associated with visual cortical surface area are feature-specific rather than generic.

In primate early visual cortices, a prominent visual feature that has an orderly cortical representation is orientation, whereas contrast and luminance do not have orderly cortical representations^15^. We therefore studied whether interindividual variability in visual cortical surface area was correlated with orientation discrimination sensitivity and orientation contextual modulation magnitude (measured through contextual illusion), and whether such correlations, if observed, generalized to contrast and luminance domains. As the discrimination sensitivity and contextual illusion magnitude of these three visual features all depend on stimulus size^6^,^7^,^8^,^9^,^10^,^11^ and involve early visual cortices^16^,^17^,^18^, they offered a varied battery of tests for our two hypotheses. We find that across healthy human adults, an increase in visual cortical surface area is associated with improved orientation discrimination sensitivity but weakened orientation contextual illusion. This anatomically associated tradeoff between discrimination sensitivity and contextual modulation magnitude, however, does not generalize to contrast or luminance perception. Computational simulations based on the scaling of intracortical connections are able to reproduce our empirical findings. Together, the converging evidence is most consistent with our second hypothesis, demonstrating a feature-specific change in the scope of visual perception from global, context-oriented to local, detail-oriented with increased visual cortical surface area.

## Results

### Tradeoff between discrimination and contextual modulation

Our hypotheses predict a tradeoff between visual discrimination sensitivity and contextual modulation magnitude that is associated with variation in visual cortical surface area. Therefore, we first investigated whether such a perceptual tradeoff existed across a large group of healthy adult participants (*N*=45), in orientation, contrast and luminance domains.

We measured discrimination sensitivity using 2-up-1-down staircase that assessed the just-noticeable feature differences between two successively presented visual stimuli^19^. We measured contextual modulation magnitude using contextual illusion stimuli where the perceived orientation or contrast, or luminance of a central stimulus was biased by the surrounding context and differed from its physical value^20^. In contextual illusions, the presence of the surrounding context not only induces illusory perception of the central stimulus but may also influence the discrimination sensitivity of the central stimulus, although the effect of the latter is less robust than the former^21^,^22^,^23^,^24^,^25^,^26^,^27^. We therefore used the former (magnitude of contextual illusion) as an indicator of contextual modulation magnitude and reported results of the later (contextual influences on discrimination sensitivity) in Supplementary Note 1. The contextual illusion magnitude, quantified as the feature difference between two physically dissimilar stimuli that appeared perceptually equal because of the presence of the surrounding context, was measured using the method of constant stimuli. For both the visual discrimination experiment and the contextual illusion experiment, we used a temporal two-alternative-forced-choice paradigm with stimuli presented at central fixation, so as to prevent the complication of spatial inhomogeneity arising from comparing stimuli across different visual field locations in spatial two-alternative-forced-choice paradigms^28^.

**Figure 1:**
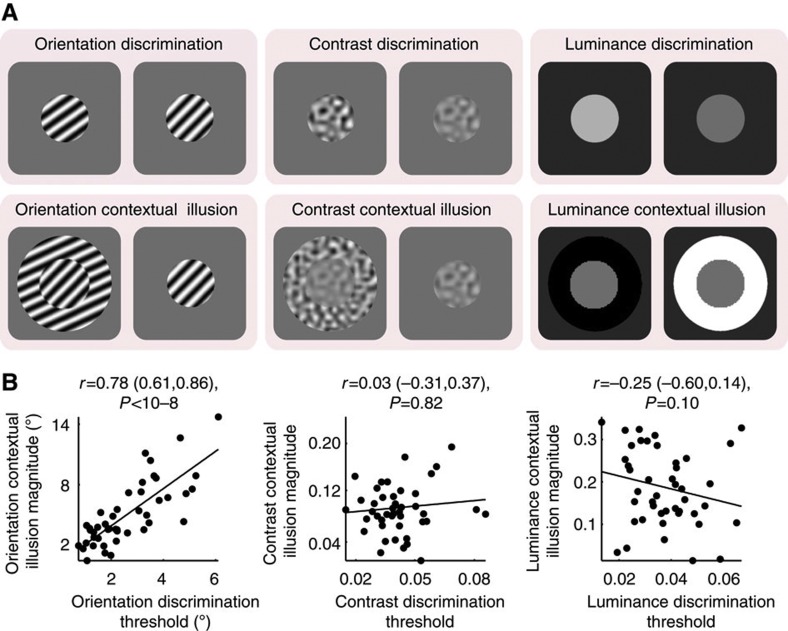
Tradeoff between visual discrimination sensitivity and contextual illusion magnitude. (**a**) Illustrated are visual stimuli used in psychophysical experiments. Visual discrimination threshold, quantified as the just-noticeable feature differences between two successively presented visual stimuli, was measured using a standard staircase procedure. Contextual illusion magnitude, quantified as the feature difference between two physically dissimilar stimuli that appeared perceptually equal because of the presence of the surrounding context, was measured using a standard method of constant stimuli. (**b**) Across participants, the orientation discrimination threshold is plotted against the orientation contextual illusion magnitude, illustrating a tradeoff between discrimination sensitivity and contextual modulation of orientation perception. For contrast and luminance perception, no such tradeoff was observed between the discrimination threshold and the contextual illusion magnitude. Each point represents a single participant (*N*=45) and the line is the best-fitting linear regression. Statistical values reflect Spearman’s *ρ* and its bootstrap confidence interval with FDR correction for multiple comparisons (*α*=0.025).

We observed substantial interindividual variability both in discrimination sensitivity^29^ and in contextual illusion magnitude^20^. Across participants, orientation discrimination threshold correlated strongly with orientation contextual illusion magnitude (Fig. 1b), indicating that individuals who were able to discriminate finer orientation differences tended to experience weaker orientation contextual modulation. However, we did not observe any significant correlation across participants between contrast discrimination threshold and contrast contextual illusion magnitude, or between luminance discrimination threshold and luminance contextual illusion magnitude (Fig. 1b). Moreover, the correlation between discrimination threshold and contextual illusion magnitude in orientation perception was significantly higher than that for contrast perception (*t*(42)=7, *P*<10^−7^) or luminance perception (*t*(42)=11, *P*<10^−13^). Thus, the interindividual tradeoff between discrimination sensitivity and contextual modulation magnitude was not a generic perceptual phenomenon, but was instead observed only in the orientation domain among the three feature domains (orientation, contrast, luminance) we studied.

### Visual cortical surface area mediates perceptual tradeoff

Next we investigated how the retinotopically defined surface area of human early visual cortices related to visual discrimination sensitivity and contextual modulation magnitude, in orientation, contrast and luminance domains. We used standard phase-encoded retinotopic mapping^30^ to delineate the part of early visual cortices (V1, V2, V3) that responded to visual field stimulation up to 8.5° eccentricity (that is, a constant area of visual field for all participants) in 20 of the original 45 participants. To test whether our results were robust against the precise spatial extent of the visual field mapped and the retinotopic mapping paradigm, we recruited another independent group of participants (*N*=20). In this second group, the part of early visual cortices that responded to visual field stimulation up to 7.2° eccentricity was delineated using standard phase-encoded retinotopic mapping^30^ and confirmed using population-receptive-field retinotopic mapping^31^. The results from the main group of participants are reported below, and the replications in the second group of participants are reported in Supplementary Note 2.

**Figure 2:**
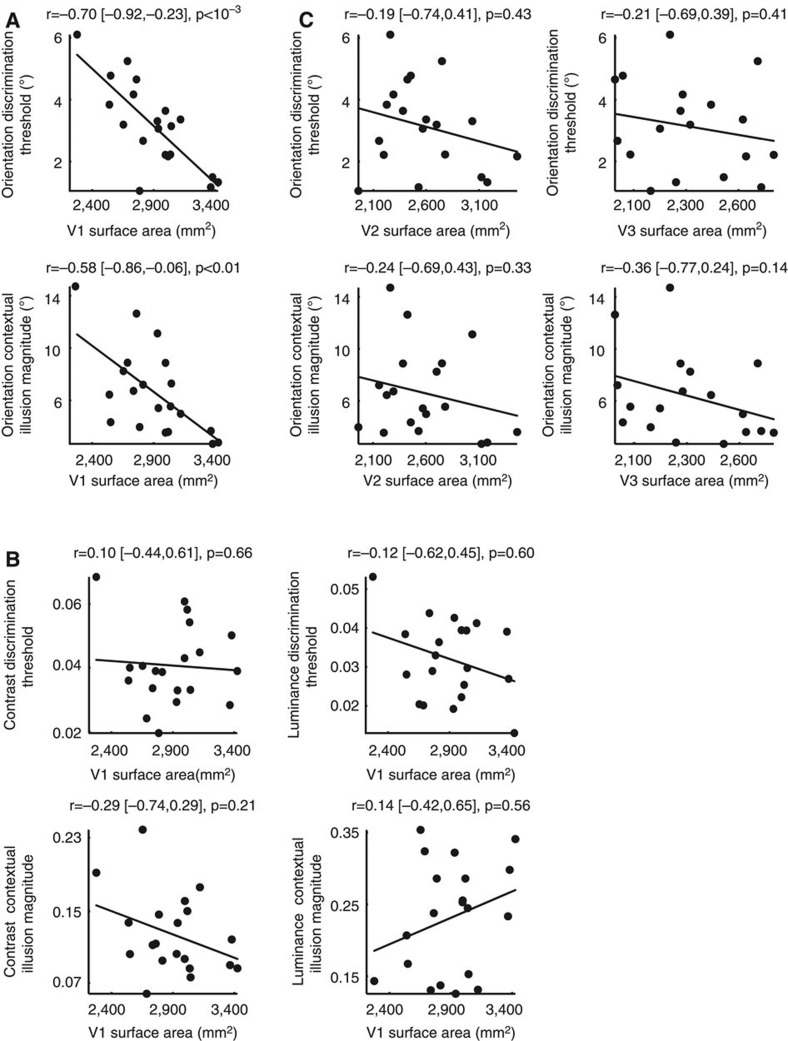
Visual cortical surface area mediates perceptual tradeoff. (**a**) Orientation discrimination threshold and orientation contextual illusion magnitude were plotted against the retinotopically defined surface area of V1, illustrating that V1 surface area negatively correlated with orientation discrimination threshold and orientation contextual illusion magnitude across participants. (**b**) The discrimination threshold and contextual illusion magnitude of contrast or luminance perception were plotted against the V1 surface area, illustrating a lack of correlation between V1 surface area and perception of contrast or luminance. (**c**) The orientation discrimination threshold and orientation contextual illusion magnitude were plotted against the surface area of V2 or V3, illustrating a lack of correlation between V2 or V3 surface area and the orientation perception. Each point represents a single participant (*N*=20) and the line is the best-fitting linear regression. Statistical values reflect Spearman’s rho and its bootstrap confidence interval with FDR correction for multiple comparisons (*α*=0.025).

We found that across participants the retinotopically mapped surface area of primary visual cortex (V1) correlated negatively with orientation discrimination threshold (Fig. 2a), suggesting that individuals with larger V1 could discriminate finer orientation differences. Conversely, individuals with larger V1 were less susceptible to orientation contextual modulation, as the surface area of V1 correlated negatively with the magnitude of orientation contextual illusion (Fig. 2a). The relationships between V1 surface area and orientation perception did not generalize to luminance perception or contrast perception. There were no significant correlations between V1 surface area and contrast discrimination threshold, contrast contextual illusion magnitude, luminance discrimination threshold, or luminance contextual illusion magnitude (Fig. 2b); and orientation perception correlated with V1 surface area significantly higher than contrast (*t*(17)=2.3, *P*<0.05) or luminance perception (*t*(17)=3.1, *P*<0.01) did. Similarly, we found that the correlations between orientation perception and visual cortical surface area were specific to V1 and did not generalize to V2 or V3 that had significantly lower correlations (Fig. 2c; V1 versus V2, *t*(17)=2.8, *P*<0.01; V1 versus V3, *t*(17)=2.4, *P*<0.05). Together, these results revealed a selective correlation between the surface area of human V1 and the scope of orientation perception (local detail-oriented versus global context-oriented) that did not generalize to the surface area of other early retinotopic cortices or to the perception of luminance and contrast.

**Figure 3:**
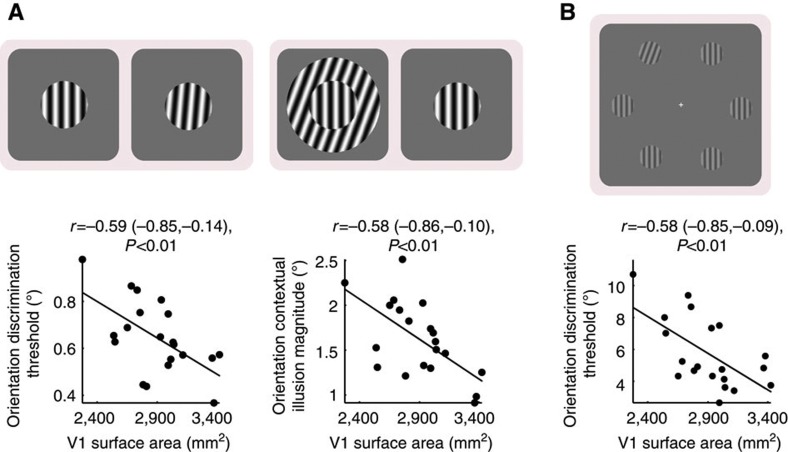
Robustness of correlation between V1 surface area and orientation perception. To test whether the correlation between V1 surface area and orientation perception was robust against the psychophysical paradigm/stimulus, we conducted additional control experiments. (**a**) Orientation discrimination threshold and orientation contextual illusion magnitude were measured using cardinally oriented stimuli in replace of the obliquely oriented stimuli used in the original experiments. The measures were plotted against V1 surface area, illustrating a negative correlation between V1 surface area and orientation discrimination threshold or orientation contextual illusion magnitude. (**b**) Orientation discrimination threshold was measured using a two-alternative-forced-choice paradigm with peripheral presentation of visual stimuli in replace of the temporal two-alternative-forced-choice paradigm with foveal presentation of visual stimuli used in the original experiments. The measure was plotted against V1 surface area, illustrating a negative correlation between V1 surface area and orientation discrimination threshold. Each point represents a single participant (*N*=20) and the line is the best-fitting linear regression. Statistical values reflect Spearman’s rho and its bootstrap confidence interval with FDR correction for multiple comparisons (*α*=0.025).

These results were replicated in the second group of participants (Supplementary Fig. S1), suggesting that the correlation between V1 surface area and orientation perception was robust to the retinotopic-mapping paradigm/stimulus used for measuring the V1 surface area. To test whether this correlation was also robust to the psychophysical paradigm/stimulus used for assessing orientation perception^32^,^33^, we conducted control experiments where we used cardinally oriented stimuli or a spatial two-alternative-forced-choice paradigm (Supplementary Note 3) to replace the obliquely oriented stimuli and the temporal two-alternative-forced-choice paradigm used in the original experiments. Under cardinally oriented stimuli, we still observed a tradeoff between orientation discrimination sensitivity and orientation contextual illusion magnitude that correlated with the V1 surface area (Fig. 3a). Similarly, we also observed a significant correlation between V1 surface area and orientation discrimination sensitivity measured using spatial two-alternative-forced-choice paradigm with peripheral presentation of visual stimuli (Fig. 3b). Thus, the correlation between the surface area of human V1 and the scope of orientation perception was a robust observation independent of the particular experimental paradigms.

Our overall findings, that interindividual increase in V1 surface area was selectively associated with a shift in orientation perception from context-oriented to detail-oriented, support our second hypothesis. It suggests that visual cortical surface area influences visual perception through the scaling of intracortical connections, and an orderly cortical representation of a feature space may be essential for the surface area of this visual cortex to influence of the perception of this visual feature. Indeed, in primate V1, orientation is orderly represented with an unbroken coverage over the cortical surface^14^. By contrast, in V2 or V3 the orientation representation (thick stripe, pale stripe) is interleaved with the colour representation (thin stripe)^34^. Moreover, neither luminance nor contrast has orderly representations in early retinotopic visual cortices^14^,^35^. These different topologies of cortical representations are likely to have different perceptual consequences when intracortical connectivity decreases with increased visual cortical surface area. Similar to orientation, ocular dominance also has an orderly cortical representation that has been observed in human primary visual cortex^36^. Our second hypothesis thus suggests that the interindividual variability in visual cortical surface area may have perceptual consequences on interocular suppression. We conducted some preliminary tests of this possibility, the details of which are described in Supplementary Note 4.

### Computational simulations reproduce empirical findings

Finally, as a proof of concept we used computational simulations to test whether the scaling of intracortical connections in a model based on the functional organization of primate early visual cortices could reproduce our empirical observations. A great number of models exist that capture the characteristics of visual discrimination and contextual modulation, including ‘structural’ models based on cortical circuits^37^,^38^, ‘functional’ models based on neural computations, such as normalization^39^, and ‘statistical’ models based on natural scene statistics^40^. As our question of interest was intracortical circuit scaling, we used ‘structural’ models and, in particular, neural field models that allow interpretations in terms of human perception^41^ and retain the explanatory power of large-scale circuit models, but with many fewer free parameters^37^,^38^,^42^.

**Figure 4:**
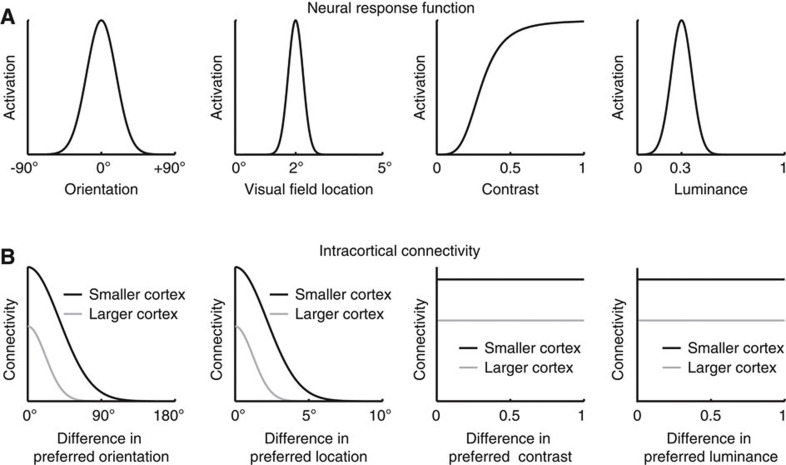
Structure of neural field model. We built a neural field model based on the established functional organization of primate early visual cortices to simulate how the scaling of intracortical circuits with visual cortical surface area influenced the perception of different visual features. We modelled the processing of four basic visual features—orientation, contrast, luminance and visual field location. (**a**) In accordance with the empirical literature, model neurons responded to contrast in monotonic function, and orientation, luminance or visual field location in Gaussian functions. (**b**) The orientation or visual field location preference of the model neurons was laid out in an orderly fashion where intracortical connectivity covaries with the similarity in feature preference between connected neurons; by contrast, the contrast or luminance preference of the model neurons was laid out in a randomized fashion without systematic relationships between intracortical connectivity and neural feature preference. As a result, for the processing of orientation or visual field location, the decrease of intracortical connectivity with increased visual cortical surface area predominantly influenced the connectivity between model neurons with similar feature preference, whereas for the processing of contrast or luminance the influence on the connectivity between model neurons with similar feature preference was counter-balanced by that between model neurons with opposite feature preference.

We modelled the processing of four basic visual features—orientation, contrast, luminance and visual field location. In accordance with the empirical literature, model neurons code contrast in monotonic response function^43^, and orientation, luminance or visual field location in Gaussian response functions^44^,^45^ (Fig. 4a). The details of the model are described in Supplementary Note 5. In the model visual cortex, orientation or visual field location had an orderly cortical representation, where the distance in cortical space reflected the distance in feature space, and the intracortical connectivity covaried with the similarity in feature preference between connected neurons (Fig. 4b). As a result, the scaling of intracortical connections predominantly influenced the connectivity between neurons with similar feature preference. By contrast, luminance or contrast did not have such an orderly cortical representation in the model visual cortex and the intracortical connectivity remained independent of the neural feature preference^15^ (Fig. 4b). Consequently, the scaling of intracortical connections equally affected the connectivity between neurons with similar versus opposite feature preferences.

**Figure 5:**
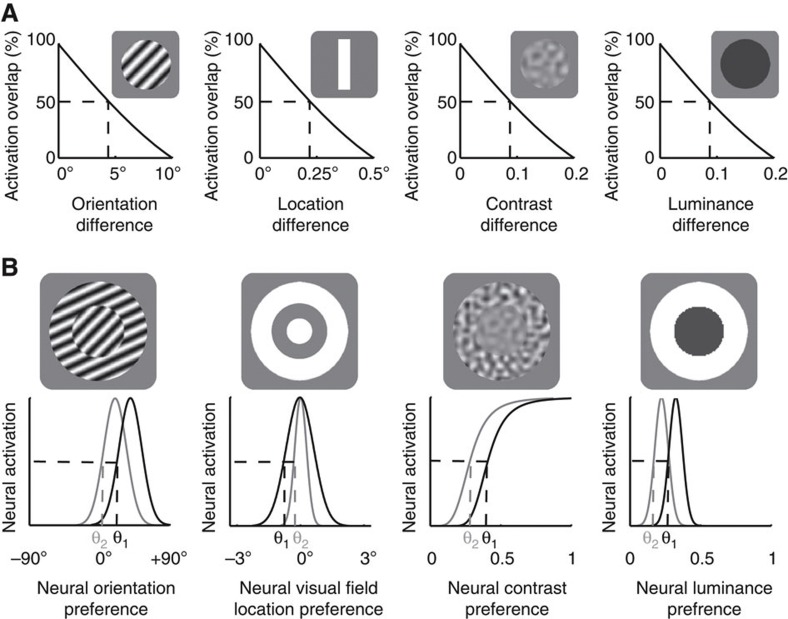
Simulations of neural field model. We simulated the model using stimuli similar to the ones used in our psychophysical experiments. (**a**) To quantify the model’s visual discrimination sensitivity, we simulated it with a set of stimuli that differed in only a single feature (orientation, contrast, luminance and visual field location). The activation pattern of the model visual cortex was compared for different stimuli along each feature dimension, where the degree of overlap in activation pattern decreased with the feature difference between stimuli. The feature difference at the threshold point where the activation overlap decreased to 50% was quantified as the model’s visual discrimination threshold. (**b**) To quantify the model’s contextual modulation magnitude, we simulated it with contextual illusion stimuli where a central circular stimulus was surrounded by a surrounding annular stimulus. The response of model neurons to the central stimulus was modulated by the response of their neighbouring neurons to the surrounding stimulus, where the inhibitory connections from neighbouring neurons caused a repulsive shift in the model’s response to the central stimulus (from black to grey line). The extent of this shift was quantified as the model’s contextual modulation magnitude.

We simulated the model using stimuli similar to the ones used in our psychophysical experiments. To quantify the model’s visual discrimination sensitivity, we simulated it with a set of stimuli that differed in only a single feature (orientation, contrast, luminance and visual field location). The activation pattern of the model visual cortex was compared for different stimuli along each feature dimension, where the degree of non-overlap in activation pattern increased with the feature difference between stimuli (Fig. 5a). Such non-overlap in activation pattern represented the model’s accuracy in discriminating these stimuli. The model’s visual discrimination threshold was quantified as the feature difference at the threshold point where the activation overlap decreased to 50% (Fig. 5a). To quantify the model’s contextual modulation magnitude, we simulated it with contextual illusion stimuli where a circular stimulus was surrounded by an annular stimulus of different orientation, contrast, luminance, or by an annular stimulus of equal feature parameters (location/size illusion). The model neurons’ response to the central stimulus was modulated by the response of their neighbouring neurons to the surrounding stimulus, where the inhibitory connections from neighbouring neurons caused a repulsive shift in the model’s response to the central stimulus^16^ (Fig. 5b). This shift in activation pattern resembled the illusory perception induced by the presence of surrounding context, and the extent of this shift was quantified as the model’s contextual modulation magnitude.

**Figure 6:**
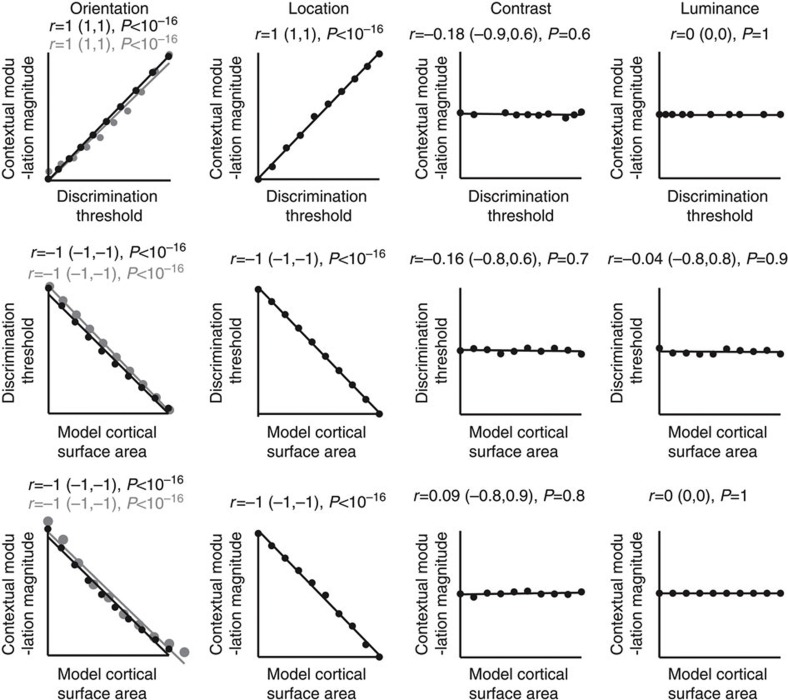
Model simulations reproduce empirical observations. We varied the independent variable in the model—the surface area of the model visual cortex, to investigate its influence on the model’s outputs—the discrimination threshold and contextual modulation magnitude. When the model visual cortex expanded in surface area, the cortical representation of visual field location expanded accordingly, and the cortical representation of orientation expanded as well through an increase in either the number or the size of orientation hypercolumns (the simulation results of which were marked with black and grey colour, respectively). Mirroring the empirical observations, the model simulations revealed a tradeoff between discrimination sensitivity and contextual modulation magnitude that correlated with the surface area of the model visual cortex. This tradeoff and its correlation with the model visual cortical surface area were only evident in the domain of orientation or visual field location and were not observed in the domain of luminance or contrast. Each point represents a single simulation (*N*=10) and the line is the best-fitting linear regression. Statistical values reflect Spearman’s rho and its bootstrap confidence interval with FDR correction for multiple comparisons (*α*=0.025).

The model simulations revealed a tradeoff between discrimination sensitivity and contextual modulation magnitude that correlated with the surface area of the model visual cortex (Fig. 6). This tradeoff and its correlation with the model visual cortical surface area, however, were only evident in the domain of orientation or visual field location and were not observed in luminance or contrast domain (Fig. 6). These results mirror our empirical findings. They suggest that visual cortical surface area selectively influences the perception of visual features that have orderly cortical representations through the scaling of intracortical connections. For these visual features, the scaling of intracortical connections directly influences the connectivity between neurons with similar feature preference, whereas for visual features without orderly cortical representations the influence on the connectivity between neurons with similar feature preference is counter-balanced by that between neurons with opposite feature preference (Fig. 4). As visual discrimination and contextual modulation involve interactions between features close in similarity, the scaling of intracortical connectivity with the similarity in feature preference between connected neurons may be a mechanism through which visual cortical surface area influences feature perception, as suggested by the simulation results here. Thus, as a proof of concept we show that computational modelling based on the scaling of intracortical connections with visual cortical surface area can reproduce our empirical observations.

## Discussion

Recent years have witnessed a growing interest in the correlations between macroscopic cortical anatomy and human behaviour^46^. However, the potential mechanisms through which cortical anatomy influences human behaviour remain unclear. This is in large because of the limited knowledge of the mesoscopic and microscopic architecture of most cortical areas. Moreover, using gross anatomy to coregister different structural images to a common template or using brain atlases to define the boundaries of cortical regions may fail to capture interindividual variability in the functional localization of a cortical region, which adds to the complexity of interpreting these anatomy–behaviour correlations. In contrast to most other cortical areas, early visual cortices have relatively well-defined architectures at both mesoscopic and microscopic level^14^,^15^,^44^. More importantly, the functional localization of early visual cortices can be measured precisely for each individual using retinotopic mapping^30^.

The well-defined and measurable structure of human early visual cortices provides an opportunity for exploring the mechanisms through which visual cortical surface area influences perception. Here we tested two possible mechanisms that interindividual variability in visual cortical surface area influences perception through the scaling of effective stimulus size alone or through its interaction with the scaling of intracortical circuits. Although these two hypotheses both predicted a change in the scope of visual perception from global, context-oriented to local, detail-oriented with the increases in visual cortical surface area across individuals, this change is predicted to be generic for the first hypothesis but feature-specific for the second hypothesis. Previous studies on related topics show that the retinotopically defined V1 surface area correlates positively with the sensitivity of visual field location discrimination^47^ and negatively with the magnitude of size contextual illusion^5^. These findings are consistent with both of our hypotheses and are reproduced by our computational simulations (Fig. 6). Specifically, the computational simulations that model the perception of visual field location can also explain the perception of stimulus size, as a contraction/expansion in the perceived visual field location of stimulus edge contributes to a change in the perceived size of the visual stimulus.

To explicitly test our two hypotheses, we studied whether variation in visual cortical surface area was associated with a tradeoff between sensitivity to visual details and susceptibility to visual context for perception of the same visual feature, and whether any such tradeoff was generic or feature-specific. We found that interindividual variability in the V1 surface area was associated with a feature-specific tradeoff between discrimination sensitivity and contextual modulation of orientation perception. Our findings therefore supported our second hypothesis, suggesting that an orderly cortical representation of a visual feature where the distance in cortical space reflects the distance in feature space is essential for the scaling of cortical surface area to influence the perception of this visual feature. Indeed, not all visual features have such orderly cortical representation. Rather, only a limited number can be represented in this orderly fashion^15^. Among these, a notable feature is visual orientation. The orderly cortical representation of orientation is an organizational principle observed in many mammalian species and may carry ecologically robust information^48^. Even in rodents where V1 does not exhibit a discernible orientation map (possibility because of its small size), orientation information is still coded in an orderly cortical representation where the intracortical connectivity covaries with the similarity in preferred orientation between connected neurons^44^, and sister neurons from the same ontogenetic column share similar orientation preferences^49^.

For a deeper understanding, it will be of interest for further studies to test how visual cortical surface area affects perception of other visual features. For example, in addition to orientation, another prominent visual feature that has an orderly cortical representation in human primary visual cortex is ocular dominance^36^. Our second hypothesis suggests that as the visual cortical surface area increases across individuals, the decrease of intracortical connectivity will result in weakened intracortical inhibition between neurons with opposite ocular preference. Consequently, individuals with larger V1 surface area may experience weaker interocular suppression. One behavioural measure for the degree of interocular suppression is binocular rivalry, where perception alternates between the two incompatible monocular stimuli^50^. Perceptual alternation in binocular rivalry reflects the effect of interocular suppression^50^,^51^ and may thus correlate with the V1 surface area. Interestingly, our preliminary tests of this possibility, although statistically non-significant, hint towards such a relationship (Supplementary Note 4), which provides an interesting avenue to pursue in future studies.

Of equal importance to the correlations between visual cortical surface area and perception is consideration of the interindividual perceptual variability itself. Specifically, as luminance or contrast discrimination and contextual illusion did not exhibit any correlation with visual cortical surface area, the anatomical sources that account for interindividual variability in luminance or contrast perception are of particular interest. Just as neurons in V1 are connected in an orderly fashion according to their orientation preference^15^, neurons in the retina have orderly connectivity patterns that follow their luminance and contrast selectivity^52^. It is thus possible that variability across individuals in luminance and contrast perception may partially reflect variability in retinal structure. Other than anatomical sources, interindividual differences in neurotransmitter concentration may also contribute to perceptual variability. For example, as visual discrimination sensitivity and contextual illusion magnitude may reflect neural response gain and neural response suppression that are regulated by GABA (γ-aminobutyric acid)^52^,^53^, occipital GABA concentration may explain part of the interindividual perceptual variability. Indeed, such correlations have been reported^54^,^55^. The additional or complementary sources of individual variability in visual perception therefore remain an interesting topic for future research.

As demonstrated here and elsewhere^20^,^29^, individuals differ substantially in their perception of simple visual features. It is possible that perception of complex visual images will exhibit an equally large, if not larger, degree of interindividual variability. Whether interindividual variability in high-level perception might be related to that of simple feature perception and associated with individual differences in early visual cortical surface area will be of future research interest. Here by examining the relationship between the retinotopically defined surface area of early visual cortices and the perception of different visual features, we showed that an interindividual increase in the visual cortical surface area is associated with a feature-specific perceptual shift from being strongly modulated by global visual contexts to being highly discriminative of local visual details. Moreover, we showed that neural field simulations based on the scaling of intracortical circuits could reproduce our empirical observations. Together, our results converge to suggest that visual cortical surface area selectively influences the perception of visual features with orderly cortical representations. We hope that our work provides an initial framework for future studies that further investigate how sensory cortical surface areas might serve as endophenotypes of individual sensory experiences.

## Methods

### Participants and apparatus

One group of 45 healthy volunteers (20 women, 25 men, aged 21–35 years) and a second group of 20 healthy volunteers (10 women, 10 men, aged 19–34 years), with normal or corrected-to-normal vision and no significant neurological history, took part in this study. All participants gave written informed consent. The study was approved by the UCL Ethics Committee.

Psychophysics experiments were conducted in a darkened room where the only significant source of light was provided by the calibrated cathode ray tube (CRT) computer monitor (spatial resolution=1024 × 768 pixels; refresh rate=100 Hz; viewing distance=67 cm; minimum luminance=0.51 cd m^−2^; maximum luminance=80.9 cd m^−2^). Neuroimaging experiments were conducted in a Siemens Trio 3T MRI (magnetic resonance imaging) scanner with a 32-channel head-coil. Structural MRI data were collected using T1-weighted modified driven equilibrium fourier transform (MDEFT) sequence (repetition time (TR)=7.92 ms; echo time (TE)=2.48 ms; flip angle=16°; field of view=256 × 240; 176 slices; resolution=1 × 1 × 1 mm). Functional MRI (fMRI) data were collected using a two-dimensional echo planar imaging (EPI) sequence (TR=3.06 s; matrix size=96 × 96; resolution=2.3 × 2.3 × 2 mm) for the first group of participants, and a three-dimensional multi-shot EPI sequence (TR=3.2 s; matrix size=128 × 128; resolution=1.5 × 1.5 × 1.5 mm) for the second group of participants. Visual stimuli were projected onto a screen (size=28.6 × 21.5 cm) in the back of the scanner and viewed through a mirror on the head coil (viewing distance=72 cm for the first and 85 cm for the second group of participants).

### Neuroimaging experiments

Standard phase-encoded retinotopic mapping was applied to localize V1, V2 and V3 (ref. 30). Each participant took part in two runs of polar-angle mapping and one run of eccentricity mapping. For polar-angle mapping, the stimuli were full-contrast flickered (flicker rate=4 Hz) checkerboard wedges (width=40°) rotating smoothly in clockwise or anticlockwise direction around a small fixation cross. In a single run, the stimuli rotated for 10 cycles at a speed of 20 volumes per cycle. For eccentricity mapping, the stimuli were full-contrast flickered checkerboard rings (width=7.8% of the screen length) contracting smoothly around a small fixation cross. In a single run, the stimuli contracted for 15 cycles at 15 volumes per cycle. To maintain participants’ attention, at random temporal intervals the mapping stimuli would undergo a small pattern shift for 200 ms. Participants were asked to indicate whenever this happened with a button press while keeping their eyes fixated at the central cross during the whole experiment.

The fMRI data were preprocessed in Statistical Parametric Mapping software (SPM8) using slice time correction, realignment, unwarping and coregistration to MRI structural image. Polar-angle maps and eccentricity maps were generated by applying fast fourier transform to fMRI time series of each voxel to extract its phase at the stimulation frequency. F-statistic maps indicating the significance of visual response were calculated by dividing the power at the stimulation frequency by the average power across all frequencies. Polar-angle maps were used to delineate the boundaries between different visual areas (V1, V2d, V2v, V3d and V3v). The maps were displayed on inflated cortical surfaces reconstructed using FreeSurfer, and the boundaries between different visual areas were delineated manually according to the mirror reversals in the maps. F-statistic maps were used to delineate the inner and outer edges of the retinotopically mapped part of V1, V2 and V3. The maps were thresholded at a significance level of *P*<0.05 (uncorrected). The surface area of each visual cortical region (V1, V2 and V3) was calculated in FreeSurfer by summing up the surface area of all vertices in that cortical region.

### Psychophysics experiments

Visual discrimination threshold and contextual illusion magnitude of orientation, contrast and luminance perception were measured separately in six subexperiments. To disentangle the effects associated with different visual features, we used visual stimuli that each contained information associated with only a single feature. Specifically, uniform gray stimuli that contained no contrast and no orientation information were used in the luminance experiment; low-pass filtered white noise stimuli that contained no orientation information and had constant average luminance were used in the contrast experiment; sinusoidal grating stimuli (spatial frequency=1.5 cycles per visual degree) that had constant contrast (full contrast) and constant average luminance were used in the orientation experiment. The stimuli were presented on a uniform gray background whose luminance was 50% of the monitor maximum luminance for orientation and contrast experiments, and 20% of the monitor maximum luminance for luminance experiment. To minimize confounding factors that may influence the anatomy–perception relationship differently for different visual features, we applied the same psychophysical paradigms in orientation, contrast and luminance experiments. Specifically, we applied standard 2-up-1-down staircase procedure to measure visual discrimination threshold and standard method of constant stimuli to measure contextual illusion magnitude^19^. Visual discrimination threshold and contextual illusion magnitude were both measured with temporal forced-choice paradigms where stimuli were centred at fixation.

To measure visual discrimination threshold, two circular stimuli (diameter=1.5 visual degree) were presented in succession where one stimulus had a constant feature value (45° for orientation experiment, 40% for contrast experiment, 50% of the monitor maximum luminance for luminance experiment) and the other a variable feature value. The interval (first or second), where the stimulus with constant feature value appeared, was randomized. Participants made an unspeeded forced choice regarding whether the second stimulus, compared with the first one, was rotated clockwise or anticlockwise (orientation discrimination), had higher or lower contrast (contrast discrimination), or had higher or lower luminance (luminance discrimination). The duration of each stimulus was 300 ms and the interstimulus interval was 500 ms. The orientation, contrast or luminance difference between the two successively presented stimuli was varied in standard 2-up-1-down staircase fashion that assessed the threshold value at which the discrimination performance converged to 70.7% correct^19^. Specifically, two consecutive correct answers led to the feature difference in the next trial being one step lower than in the previous trials, whereas one incorrect answer led to an increase in the feature difference. The experiment stopped after 18 reversals, and the discrimination threshold was calculated as the feature difference averaged over the last 10 reversals. Throughout the staircase procedure, the staircase step size was 0.25° for orientation discrimination, 0.5% for contrast discrimination and 0.25% of the monitor maximum luminance for luminance discrimination.

To measure contextual illusion magnitude, two central circular stimuli (diameter=1.5 visual degree), one with and one without surrounding annular context (inner diameter=1.5 visual degree; outer diameter=6 visual degree), were presented in succession. The duration of each stimulus was 300 ms and the interstimulus interval was 500 ms. The interval (first or second), where the surrounding context appeared, was randomized but counterbalanced. Participants made an unspeeded forced choice regarding whether the central stimulus in the second interval, compared with the one in the first interval, was rotated clockwise or anticlockwise (orientation illusion), had higher or lower contrast (contrast illusion), or had higher or lower luminance (luminance illusion). Before the experiment, each participant performed four trials in which they manually adjusted the orientation, contrast or luminance of the central stimulus presented in isolation till it matched the perceived orientation, contrast or luminance of the central stimulus presented in the surrounding context. In the subsequent experiment, the orientation, contrast or luminance of the central stimulus presented in surrounding context was kept constant, while that of the central stimulus presented in isolation was varied around this previously measured point of perceptual equality for seven different values. In each experiment, a total of 112 trails (16 trails per feature value) were taken to produce a psychometric curve fitted with logistic function. The contextual illusion magnitude was quantified as the feature difference between the two central stimuli at the 50% threshold point of the psychometric curve where they appeared perceptually equal. In the orientation experiment (tilt illusion), the surrounding context was tilted 15° from the central stimulus. In the contrast experiment (contrast–contrast illusion), the surrounding context had a contrast of 100% and the central stimulus a contrast of 40%. In the luminance experiment (simultaneous brightness contrast illusion), the surrounding context was always present, and it was black in one interval, whereas white in the other.

### Computational simulations

On the basis of the functional organization of primate early visual cortices, we built a one-dimensional single-layer neural field model that described how the intracortical circuits modulated the spatiotemporal dynamics of neural activity^38^,^41^,^42^. The model is a computational simplification and a one-dimensional projection of a two-dimensional cortical sheet. The independent variable in the model is the surface area (width) of model visual cortex, which is proportional to the number of model neurons^56^. The model gives output of discrimination threshold and contextual modulation magnitude. The details of the model are described in Supplementary Note 5.

## Author contributions

C.S., D.S.S. and G.R. designed the overall study and wrote the paper. C.S. and D.S.S. collected and analysed the fMRI data. C.S. collected and analysed the psychophysics data and designed and built the computational model.

## Additional information

**How to cite this article:** Song, C. *et al.* Variability in visual cortex size reflects tradeoff between local orientation sensitivity and global orientation modulation. *Nat. Commun.* 4:2201 doi: 10.1038/ncomms3201 (2013).

## Supplementary Material

Supplementary Information Supplementary Figure S1, Supplementary Table S1, Supplementary Notes 1-5 and Supplementary References
