# Supplementary material for: Variability in visual cortex size reflects tradeoff between local orientation sensitivity and global orientation modulation
Source: Nat Commun. 2013 Jul 26;4:2201. doi: 10.1038/ncomms3201 (PMC3731653; doi:10.1038/ncomms3201)
Supplement: Supplementary Information — Supplementary Figure S1, Supplementary Table S1, Supplementary Notes 1-5 and Supplementary References [file ncomms3201-s1.pdf]

SUPPLEMENTARY INFORMATION

Variability in visual cortex size reflects tradeoff between local orientation sensitivity and global orientation modulation

Chen Song[1,2], Dietrich Samuel Schwarzkopf[1,2], Geraint Rees[1,2]

1. Institute of Cognitive Neuroscience, University College London, 17 Queen Sq, London WC1N 3AR, U.K.

2. Wellcome Trust Centre for Neuroimaging, University College London, 12 Queen Sq, London WC1N 3BG, U.K.

This document contains:

- Supplementary Figure S1. Replications of results in the second group of participants.
- Supplementary Table S1. Parameters in the neural field model.
- Supplementary Note 1. Contextual influences on visual discrimination sensitivity.
- Supplementary Note 2. Robustness of anatomy-perception relationship to retinotopic-mapping paradigm.
- Supplementary Note 3. Robustness of anatomy-perception relationship to psychophysical paradigm.
- Supplementary Note 4. Relationships between V1 surface area and interocular suppression.
- Supplementary Note 5. Structure and simulations of neural field model.

Supplementary Figure S1. Replications of results in the second group of participants.

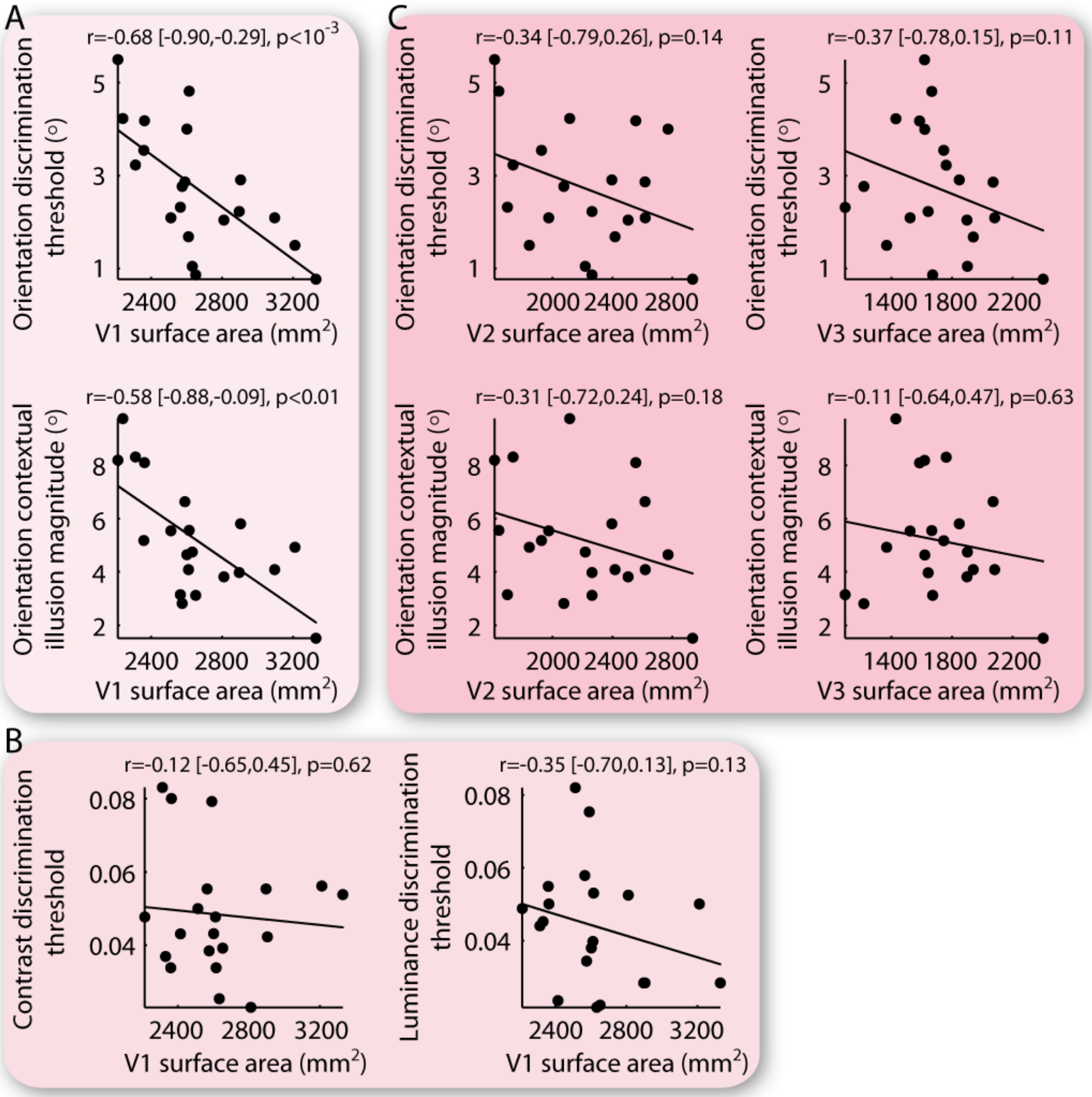

Supplementary Figure S1. Replications of results in the second group of participants. A second group of participants were recruited to test whether the relationships between visual cortical surface area and feature perception were robust to the precise spatial extent of the visual field mapped and the retinotopic mapping paradigm. The results in this second group of participants replicated these in the main group. Specifically, correlations between V1 surface area and orientation perception were observed (A) that did not generalize to the perception of luminance and contrast (B) or to the surface area of other early retinotopic cortices (C). Each point represents a single participant (N=20) and the line is the best-fitting linear regression. Statistical values reflect Spearman's rho and its bootstrap confidence interval with FDR correction for multi-comparisons (alpha=0.025).

Supplementary Table S1. Parameters in the neural field model

Parametmer	Description	Value
τ	membrane time constant	15 msec
α	transfer function gain (stepness)	15 spikes/sec/mV
υ_{low}	transfer function lower threshold	0 mV
f_{high}	transfer function higher threshold	300 spikes/sec
J_e	efficacy of lateral excitation	0.22 mV/spikes/sec
J_i	efficacy of lateral inhibition	0.09 mV/spikes/sec
σ_e	width of cortical-distance-dependent lateral excitation	2 mm
σ_i	width of cortical-distance-dependent lateral inhibition	4 mm
σ	width of orientation-preference-dependent lateral modulations	60 deg
σ_{ff}	feedforward kernel parameter	20 deg (orientation)<br>0.5 deg (location)<br>0.1 (luminance)<br>4 (contrast)

Supplementary Note 1. Contextual influences on visual discrimination sensitivity.

In a contextual illusion, the presence of a surrounding context not only induces illusory perception of the central stimulus but may also influence the discrimination sensitivity of the central stimulus[21-27]. However, whereas the contextual induction of illusory perception is robustly observed in various feature domains[20], the contextual influences on discrimination sensitivity are far less robust with, for example, some studies reporting facilitation or suppression of contrast sensitivity[25] yet others a lack of such influences[22,26,27].

In our study, we measured the contextual illusion magnitude with standard method of constant stimuli that generated a psychometric curve. This allowed us to assess not just the illusion magnitude (threshold of the psychometric function) but also the visual discrimination sensitivity under the presence of surrounding context (slope of the psychometric function). By comparing the discrimination sensitivity under the presence versus the absence of surrounding context, we were then able to evaluate the contextual influences on visual discrimination sensitivity. Since the discrimination sensitivity under the absence of surrounding context was measured through standard 2-up-1-down staircase procedure that assessed the discrimination threshold at 70.7% accuracy, the discrimination sensitivity under the presence of surrounding context was quantified as the feature difference between the 70.7% and 50% threshold points of the psychometric curve. This value reflected the minimum feature difference needed for reaching 70.7% accuracy from pure guess (50% accuracy).

We found that the discrimination sensitivities under the absence versus the presence of surrounding context were highly correlated across participants for all three visual features (Spearman's rho; orientation, $r=0.77$, $p<10^{-9}$, N=45; contrast, $r=0.47$, $p<0.001$, N=45; luminance, $r=0.42$, $p<0.005$, N=45). These correlations suggest that visual discrimination sensitivities represent robust personal traits that are relatively independent of the psychophysical paradigm and the stimulus layout.

Interestingly, while inter-individual differences in visual discrimination sensitivities were robust to the presence of surrounding context, we found that at the group level, the presence of surrounding context caused a significant decrease in orientation discrimination sensitivity (paired t-test; $t(44)=2.1$, $p<0.05$) but not in contrast (paired t-test; $t(44)=1.7$, $p=0.08$) or luminance discrimination sensitivity (paired t-test; $t(44)=1.8$, $p=0.08$). Moreover, the degree to which orientation discrimination sensitivity decreased under contextual stimulation correlated across participants with the magnitude of orientation contextual illusion, regardless of whether the baseline inter-individual difference in orientation discrimination sensitivity was regressed out (Spearman's rho; $r=0.44$, $p<0.005$, N=45) or not (Spearman's rho; $r=0.58$, $p<10^{-4}$, N=45). Such contextual modulations of visual discrimination sensitivities are consistent with previous reports that orientation sensitivity loss accompanies and covaries with orientation contextual illusion[21-23] whereas contrast sensitivity can remain unaffected by spatial contexts[22,26,27]. The consistency between our results and previous reports demonstrates the validity of our measures of visual discrimination sensitivity and contextual illusion magnitude.

Supplementary Note 2. Robustness of anatomy-perception relationship to retinotopic-mapping paradigm.

To test whether the correlation between V1 surface area and orientation perception was robust to the precise spatial extent of the visual field mapped and the retinotopic mapping paradigm, we recruited a second group of participants (N=20). In this group, the part of early visual cortices that responded to visual field stimulation up to 7.2 degrees eccentricity was delineated using standard phase-encoded retinotopic mapping[30] and confirmed using population-receptive-field retinotopic mapping[31].

In population-receptive-field (pRF) mapping, the stimuli were full-contrast flickered (flicker rate: 6Hz) checkerboard bars (width: 1.8 visual degree) oriented at one of the four orientations (horizontal, vertical, 45 degree, 135 degree) and moving along the corresponding orthogonal direction (north/south for horizontal bar, west/east for vertical bar, northwest/southeast for 45 degree bar, northeast/southwest for 135 degree bar). In a single run, the stimuli were presented for 8 cycles at a speed of 16 volumes/cycle (one visual field position per volume, each visual field position repeated twice per run). A blank screen was inserted into the last 1/4 period of the 2nd, 4th, 6th, and 8th cycle to provide a baseline condition that increased the accuracy of the measurement. To maintain participants' attention, the central fixation cross would turn from red to blue for 80 ms at random temporal intervals, and participants were asked to indicate whenever this happened with a button press while maintaining fixation during the whole experiment. To generate polar-angle maps and eccentricity maps, fMRI time series of each voxel were fitted with a two-dimensional Gaussian function multiplied by the stimulus position function and convolved with standard hemodynamic response function[57,58]. The center of the two-dimensional Gaussian function indicated the visual field location that the voxel responded strongest to, from which the polar-angle maps and eccentricity maps were calculated[31].

The relationships between visual cortical surface area and orientation, luminance, or contrast perception that we observed in the main group of participants (Fig. 2) were replicated in this second group of participants (Supplementary Fig. S1). Specifically, orientation discrimination threshold and orientation contextual illusion magnitude correlated significantly with the surface area of V1 (Supplementary Fig. S1A) but not V2 or V3 (Supplementary Fig. S1C), whereas no significant correlation was observed between contrast or luminance discrimination threshold and V1 surface area (Supplementary Fig. S1B). These replications suggested that our findings were robust to both the retinotopic mapping paradigm and the precise spatial extent of the visual field mapped.

Supplementary Note 3. Robustness of anatomy-perception relationship to psychophysical paradigm.

To test whether the significant correlation between V1 surface area and orientation discrimination sensitivity was robust to the psychophysical paradigm, we conducted control experiments where we used a spatial two-alternative-forced-choice paradigm to replace the temporal two-alternative-forced-choice paradigm used in the original experiments. In contrast to the temporal forced-choice paradigm where participants could use changes in the locations of grating stripes as cues for orientation judgments, the spatial forced-choice paradigm involved orientation comparisons across stimuli at different visual field locations and thereby avoided confounding orientation judgments with location judgments.

In the spatial two-alternative-forced-choice paradigm, six cardinally oriented sinusoidal gratings (contrast: 20%, spatial frequency: 2.2 cycles per visual degree, diameter: 2.8 visual degree) were presented in a circular fashion (eccentricity: 6.9 visual degree) around a central fixation cross. On each trial, the gratings were presented twice with each presentation lasting 200ms and the inter-presentation-interval lasting 500ms. In one of the two presentation intervals, the six gratings were exactly identical, but in the other interval, one of the six gratings differed from the rest by a slightly different orientation. The temporal interval and the spatial position of the “pop-out” grating varied randomly between trials. Participants made an unspeeded forced-choice judgment regarding which presentation interval contained the pop-out grating. Notably, although the judgment was two-interval-forced-choice, participants were comparing the orientation of the gratings presented at different spatial locations rather than at different temporal intervals. The orientation difference between the pop-out grating and the rest of the gratings was varied in standard 2-up-1-down staircase fashion that assessed the discrimination threshold at which the accuracy converged to 70.7% correct[19]. Two consecutive correct answers led to the orientation of the pop-out grating in the next trial being one step closer to the orientation of the other gratings, whereas one incorrect answer led to an increase in the orientation difference. The experiment stopped after 18 reversals, and the orientation discrimination threshold was calculated as the orientation difference averaged over the last 10 reversals.

Supplementary Note 4. Relationships between V1 surface area and interocular suppression.

As a preliminary test, we measured the degree of interocular suppression using binocular rivalry and continuous flash suppression in a subset (N=13) of participants. We used the paradigm of continuous flash suppression to measure the stimulus contrast needed for breaking interocular suppression, and the paradigm of binocular rivalry to measure the perceptual duration needed for breaking interocular suppression. To test whether these measures of interocular suppression were robust against the experiment stimuli, we ran separate experiments where the testing stimuli were left/right-oriented gratings and house/face, respectively.

In continuous flash suppression experiments, a 10-Hz stream of full-contrast Mondrian patterns and a low-contrast testing stimulus were separately presented to the two eyes. The eye (left or right) where the testing stimulus appeared was randomized. Participants made an unspeeded forced-choice response regarding whether the testing stimulus was left-oriented or right-oriented (grating experiment), or was house or face (house/face experiment). In each trial, the stream of Mondrian patterns was presented for 400 ms, and the testing stimulus was presented for 200ms (appeared 100ms after onset of Mondrian patterns, disappeared 100ms before offset of Mondrian patterns). The contrast of the testing stimulus was varied in a standard 2-up-1-down staircase fashion that assessed the threshold value at which the performance converged to 70.7% correct[19]. Specifically, two consecutive correct answers led to the testing stimulus contrast in the next trial being one step lower than in the previous trials, whereas one incorrect answer led to an increase in the testing stimulus contrast. The experiment stopped after 18 reversals, and the stimulus contrast needed for breaking interocular suppression was calculated as the testing stimulus contrast averaged over the last 10 reversals. Throughout the staircase procedure, the staircase step size was 2%.

In binocular rivalry experiments, a left-oriented and a right-oriented grating (grating experiment), or a house and a face stimulus (house/face experiment), were separately presented to the two eyes. The stimulus contrast was 50% and the eye-of-presentation was counter-balanced across runs. In a single run, the stimuli were presented for 100 seconds, during which participants tracked their perception by pressing the corresponding buttons whenever a perceptual alternation occurred. Each participant took part in four runs of grating experiment, and four runs of house/face experiment. The perceptual duration needed for breaking interocular suppression was calculated as the reciprocal of perceptual alternation rate.

Across participants, the stimulus contrast needed for breaking interocular suppression in the grating experiment correlated with that in the house/face experiment (Spearman's rho; $r=0.60$, $p<0.05$, N=13). Similarly, the perceptual duration needed for breaking interocular suppression in the grating experiment correlated with that in the house/face experiment (Spearman's rho; $r=0.74$, $p<0.005$, N=13). These

correlations suggest that our measures of interocular suppression represent stimulus-independent personal traits.

However, while our measures of interocular suppression exhibited robust inter-individual variability, we found that the measures were strongly biased by binocular asymmetry and monocular preference[59-62]. For example, in one participant, the stimulus contrast needed for breaking interocular suppression was 5% for left eye but 40% for right eye. Such bias introduced extra noise and made it hard to disentangle whether the inter-individual perceptual variability reflected the effect of interocular suppression, or reflected nuisance factors such as the degree of binocular asymmetry and monocular preference. This low ratio of signal-to-noise rendered it difficult to test the relationships between visual cortical surface area and interocular suppression.

Nevertheless, under this low signal-to-noise ratio and in our small group of participants (N=13), V1 surface area still exhibited a non-significant negative correlation with the stimulus contrast (Spearman's rho; r=-0.24, p=0.43, N=13) and perceptual duration (Spearman's rho; r=-0.22, p=0.47, N=13) needed for breaking interocular suppression. These preliminary results show that individuals with larger V1 surface area tend to experience weaker interocular suppression, although the results in this small sample did not reach statistical significance. They provide some further support for our second hypothesis, suggesting that visual cortical surface area selectively influences the perception of visual features that have orderly cortical representations through the scaling of intracortical circuits.

Supplementary Note 5. Structure and simulations of neural field model.

Based on the functional organization of primate early visual cortices, we built a one-dimensional single-layer neural field model that described how the intracortical circuits modulated the spatiotemporal dynamics of neural activity[38,41,42]. The activity of model neuron at time t and cortical location x is described by differential equation:

$$\tau \frac{\partial v(x,t)}{\partial t} = -v(x,t) + s(x,t) + \int w_{xy} f[v(y,t)]dy$$

, where $v(x,t)$ denotes the membrane potential, τ the membrane time constant, $-v(x,t)$ the self-decay to resting membrane potential, $s(x,t)$ the feedforward activity, y the cortical locations of other neurons, $v(y,t)$ the membrane potential of other neurons, and w_{xy} the connectivity between the neuron at cortical location x and other neurons at location y. The axonal response function $f(v)$ describes neurons' firing rates given their membrane potentials. It is represented by a linear function imposing lower and higher thresholds[38]:

$$f(v) = \min(\alpha \max(v - v_{low}, 0), f_{high})$$

The firing rate is zero for membrane potentials below the threshold v_{low} and grows linearly with gain α above threshold, but reaches ceiling of f_{high} to avoid implausible high firing rate.

The connectivity w_{xy} between neurons at cortical locations x and y is dependent on both the cortical distance between the two neurons and the difference in their preferred orientation[38,44]:

$$w_{xy} = (J_e e^{-\frac{(x-y)^2}{2\sigma_e^2}} - J_i e^{-\frac{(x-y)^2}{2\sigma_i^2}}) e^{-\frac{(\theta_x - \theta_y)^2}{2\sigma^2}}$$

, where $(x-y)$ denotes the cortical distance between the two neurons, $(\theta_x - \theta_y)$ denotes the difference in preferred orientation between the two neurons, J_e (J_i) represents the efficacy of cortical-distance-dependent lateral excitation (inhibition), σ_e (σ_i) represents the width of cortical-distance-dependent lateral excitation (inhibition), and σ represents the width of orientation-preference-dependent lateral modulations.

The model neurons respond to four different visual features - orientation, visual field location, contrast, and luminance - with monotonic response function for contrast and Gaussian response function for the rest. The activity at the input level (the feedforward activity) $s(x,t)$ is purely dependent on the model neurons' response functions and the stimulus feature. When the feature of interest is orientation, luminance, or visual field location, the feedforward activation of the model neuron is described by Gaussian function[44,45]:

$$s(x,t) = e^{-\frac{(\theta_x - \phi)^2}{2\sigma_{ff}^2}}$$

, where θ_x denotes the "preferred" orientation, luminance, or visual field location of the neuron that evokes its maximum response, ϕ denotes the stimulus orientation, luminance, or visual field location,

and σ_{ff} denotes the width of feedforward kernel that represents the neuron's default response range. When the feature of interest is contrast, the feedforward activation of the model neuron is described by Naka-Rushton equation[43]:

$$s(x,t) = \frac{\phi^{\sigma_{ff}}}{\phi^{\sigma_{ff}} + \theta_x^{\sigma_{ff}}}$$

, where θ_x denotes the "preferred" contrast of the neuron that evokes 50% of its maximum response, ϕ denotes the stimulus contrast, and σ_{ff} denotes the slope of feedforward kernel that represents the neuron's default response range.

Supplementary Table S1 contains a summary of the model parameters and their values that are chosen in accordance with empirical data or with previous literature[38,63,64]. In particular, the width of lateral inhibition σ_i is modeled to be larger than the width of lateral excitation σ_e[64], and both are modeled to be on the scale of a few millimeters[63].

We simulated the model with stimuli similar to the ones used in psychophysical experiments. At the input level, each model neuron's activity depends on its own response function and the stimulus feature. This input activation is modulated by the neuron's connectivity with other neurons and the activity of other neurons, which gives the output activation. To quantify the model's visual discrimination sensitivity, we simulated it with a set of stimuli that differed in only a single feature (orientation, contrast, luminance, visual field location). For different stimuli along each feature dimension, we calculated the degree of overlap in their evoked neural activation pattern:

$$y_{12} = \sum_x \frac{v_1(x) - v_2(x)}{0.5 * (v_1(x) + v_2(x))}$$

, where $v_1(x)$ and $v_2(x)$ denote the output activation of model neuron at cortical location x evoked by stimulus one and two, respectively. This degree of activation overlap represented the model's accuracy in discriminating these stimuli. It was plotted against the feature difference between stimuli, and the feature difference at the threshold point where the activation overlap decreased to 50% was defined as the model's visual discrimination threshold. To quantify the model's contextual modulation magnitude, we simulated it with contextual illusion stimuli where a circular stimulus was surrounded by an annular stimulus of different orientation, contrast, luminance, or by an annular stimulus of equal feature parameters (location/size illusion). The response of model neurons to the central stimulus was modulated by the response of their neighboring neurons to the surrounding stimulus, where the inhibitory connections from neighboring neurons caused a repulsive shift in the model's response to the central stimulus. This shift in activation pattern resembled the illusory perception induced by the presence of surrounding context. We quantified the model's contextual modulation magnitude as the extent of this shift, $(\theta_2 - \theta_1)$, where θ_2 (θ_1) denotes the "preferred" orientation, contrast, luminance, or visual field location of the model neurons whose responses are 50% of the maximum population response under the presence (absence) of surrounding context (Fig. 5B).

Supplementary References

57. Glover, G. H. Deconvolution of impulse response in event-related bold fMRI. Neuroimage 9, 416–429 (1999).

58. Friston, K. J. et al. Event-related fMRI: characterizing differential responses. Neuroimage 7, 30–40 (1998).

59. Leat, S. J. & Woodhouse, J. M. Rivalry with continuous and flashed stimuli as a measure of ocular dominance across the visual field. Perception 13, 351–357 (1984).

60. Mueller, T. J. & Blake, R. A fresh look at the temporal dynamics of binocular rivalry. Biological cybernetics 61, 223–232 (1989).

61. Ehrenstein,W. H., Arnold-Schulz-Gahmen, B. E. & Jaschinski,W. Eye preference within the context of binocular functions. Graefes. Arch. Clin. Exp. Ophthalmol. 243, 926–932 (2005).

62. Kloke, W. B. & Jaschinski, W. Individual differences in the asymmetry of binocular saccades, analysed with mixed-effects models. Biological Psychology 73, 220–226 (2006).

63. Gilbert, C. D. & Wiesel, T. N. Columnar specificity of intrinsic horizontal and corticocortical connections in cat visual cortex. Journal of Neuroscience 9, 2432–2442 (1989).

64. Kerlin, A. M., Andermann, M. L., Berezovskii, V. K. & Reid, R. C. Broadly tuned response properties of diverse inhibitory neuron subtypes in mouse visual cortex. Neuron 67, 858–871 (2010).
